# Link between Monkeypox Virus Genomes from Museum Specimens and 1965 Zoo Outbreak

**DOI:** 10.3201/eid3004.231546

**Published:** 2024-04

**Authors:** Michelle Hämmerle, Aigerim Rymbekova, Pere Gelabert, Susanna Sawyer, Olivia Cheronet, Paolo Bernardi, Sébastien Calvignac-Spencer, Martin Kuhlwilm, Meriam Guellil, Ron Pinhasi

**Affiliations:** University of Vienna, Vienna, Austria (M. Hämmerle, A. Rymbekova, P. Gelabert, S. Sawyer, O. Cheronet, P. Bernardi, M. Kuhlwilm, M. Guellil, R. Pinhasi);; Helmholtz Centre for Infection Research, Greifswald, Germany (S. Calvignac-Spencer);; University of Greifswald, Greifswald (S. Calvignac-Spencer)

**Keywords:** Monkeypox virus, mpox, Pongo, Genomics, Phylogeny, Disease Outbreaks, Zoonosis, Rotterdam, the Netherlands, Germany, Austria, viruses, orangutan

## Abstract

We used pathogen genomics to test orangutan specimens from a museum in Bonn, Germany, to identify the origin of the animals and the circumstances of their death. We found monkeypox virus genomes in the samples and determined that they represent cases from a 1965 outbreak at Rotterdam Zoo in Rotterdam, the Netherlands.

Monkeypox virus (MPXV) (*Orthopoxvirus* genus, *Poxviridae* family), which causes mpox, is a large double-stranded DNA zoonotic virus first identified in 1958 in macaque primates (*1*). The first human case was reported in 1970, and recent outbreaks have attracted worldwide public attention (*1*). The 2022 outbreak has been one of the largest documented and affected numerous countries around the globe (*1*).

MPXV is known to infect chimpanzees, one of the nonhuman great ape species (*2*). The past 3 decades that great ape–infecting viruses have been studied has provided insight into the coevolution of these viruses and their hosts, and sometimes the origins of other important human pathogens, such as herpes simplex virus 2 (*3*). Museomics, which uses DNA from museum specimens for genomic studies, complements the study of contemporary wild populations because viral DNA has been detected in museum (*4*) and archeological specimens (*5*).

We report findings related to 4 orangutan (*Pongo* sp*.*) specimens that came to the zoologic research museum Alexander Koenig in Bonn, Germany, in 1965 and that were originally reported to be from wild animals from Sumatra. We extracted DNA from the orangutan teeth, built genomic libraries (Appendix Figure 1), performed shotgun sequencing, and used a hybridization capture bait set targeting various DNA viruses.

Two of the specimens showed sufficient endogenous DNA content to validate their taxonomic assignment to Sumatran orangutans (*Pongo abelii*) genomically (Appendix Figure 2). Our analysis found low levels of human contamination (0.7%–1.1%) and short insert sizes consistent with degraded DNA but no deamination patterns typical for ancient DNA (Appendix). We conducted taxonomic classification of the captured data by using Kraken2 (https://github.com/DerrickWood/kraken2), which revealed the presence of MPXV.

MPXV is likely bound to reservoir species normally distributed throughout Africa (*6*). Because this virus has occasionally spread out of Africa, we further investigated the origin and history of the MPXV-positive orangutans. We requested, and the museum provided, a letter from the wildlife trader in the Netherlands who sold the specimens to the museum in 1965. The letter stated that the specimens originated from captive zoo animals from 1964, rather than from wild animals from Sumatra. The letter did not specify from which zoo the animals were obtained.

We then mapped the reads to a MPXV genome (GenBank accession no. KJ642614) (Appendix Figures 3, 4) from a 1965 outbreak in the Rotterdam Zoo, Rotterdam, the Netherlands. This genome was the best match and very close in age to the animals we tested. Sample MAM1965–0547 yielded the best results, showing 19.12 mean depth of coverage (Table). For the 3 other specimens, we obtained 9.57-fold, 0.03-fold, and 2.81-fold mean genome coverage.

**Table:** Relevant mapping statistics of MPXV genomes from the museum orangutan specimens from Europe when mapped to the genome responsible for the MPXV zoo outbreak in Rotterdam, the Netherlands, 1965

Sample	No. sequenced reads	Mapped reads no. duplicates, MQ>30	Mean mapping quality	Mean fragment length, bp	Mean coverage depth, + SD						
						% Coverage				Frequency first base	
						1×	5×	10×		C to G	G to A
MAM1965-547	3,139,078	27,482	36.39	125.6	19.12 + 10.60	98.73	96.8	81.85		0.023	0
MAM1965-545	1,492,386	12,963	36.6	135.68	9.57 + 5.11	98.56	84.73	38.83		0.024	0
MAM1965-544	151,272	67	34.35	64.36	0.03 + 0.24	1.812	0	0		0.143	0
MAM1965-546	270,634	4,616	36.28	108.89	2.81 + 2.62	78.78	21.48	1.54		0.014	0

*MPXV, monkeypox virus; MQ, mapping quality.

MPXVs were first identified from outbreaks in facilities housing nonhuman primates in the 1950s and 1960s. Genomes of isolates derived from those outbreaks have since been sequenced by other researchers, enabling us to investigate the potential ties of our specimens to specific outbreaks by using phylogenetic analyses. The MPXV genomes from the museum orangutans fall into clade IIa and were closely related to the genome derived from the 1965 Rotterdam Zoo outbreak (Figure; Appendix Figure 5); only 2 mutations were identified between the genomes sequenced in this study and the ones from 1965 (Appendix). The Rotterdam Zoo outbreak severely affected orangutans kept at the facility, and 6 of 10 infected animals died (*7*). Those orangutans were possibly infected by an animal that had previously been in contact with other MPXV-infected monkeys (*6*). Given the concordance of the dates and circumstances, combined with the genetic evidence, we are confident that we identified some of those animals within our museum specimens. This case is unusual because we were able to tie nonhuman great ape museum specimens to a specific outbreak. The genome isolated in 1965 and the ones obtained from dry specimens stored for >50 years are almost identical. 

**Figure:**
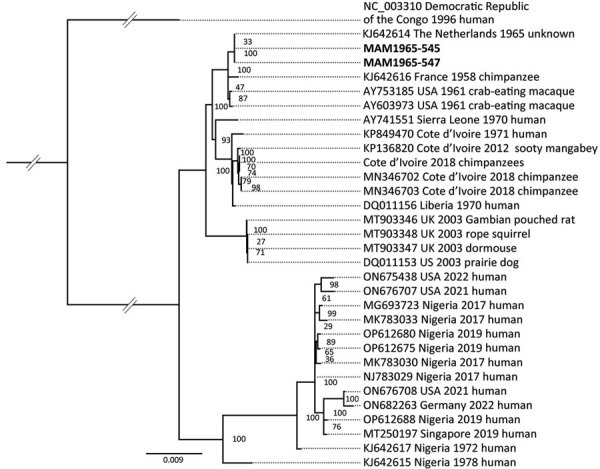
Maximum-likelihood phylogeny tree showing the close relation between MPXV genomes from museum orangutan samples from Germany (bold text), which fall into clade IIa, to the genome derived from the MPXV zoo outbreak in Rotterdam, the Netherlands, 1965. The phylogeny tree is rooted on the outgroup genome (GenBank accession no. NC_003310) from clade I with the museum orangutan genomes MAM1965–545 and MAM1965–547. The consensus sequences for the ancient sequences are based on a mapping to the Rotterdam genome. The final single-nucleotide polymorphisms alignment length was 138,240 bp. The collapsed node contains genomes from *Pan troglodytes verus* from Cote d’Ivoire (GenBank accession nos. MN346690, MN346692, MN346694–8, MN346700–1).

Our work linking the MPXV infection of those orangutans to a specific outbreak further highlights the importance of museum specimens to the study of virus diversity and evolution. Several human viruses were first discovered in captive nonhuman primates. Human respiratory syncytial virus was first identified in 1956 in captive chimpanzees (*8*,*9*). If natural history collections have regularly acquired specimens from such outbreaks and we can identify them in their records, such specimens could represent not only a treasure trove of biodiversity (*10*) but also an alternative source of pathologic specimens and infectious agent genomic material.

Appendix Additional information about link between monkeypox virus genomes from orangutan museum specimens and 1965 zoo outbreak, Rotterdam, the Netherlands.
