# Supplementary material for: Link between Monkeypox Virus Genomes from Museum Specimens and 1965 Zoo Outbreak
Source: Emerg Infect Dis. 2024 Apr;30(4):815–7. doi: 10.3201/eid3004.231546 (PMC10977839; doi:10.3201/eid3004.231546)
Supplement: Appendix — Additional information about link between monkeypox virus genomes from orangutan museum specimens and 1965 zoo outbreak, Rotterdam, the Netherlands. [file 23-1546-Techapp-s1.pdf]

Article DOI: https://doi.org/10.3201/eid3004.231546

EID cannot ensure accessibility for supplementary materials supplied by authors. Readers who have difficulty accessing supplementary content should contact the authors for assistance.

Link between Monkeypox Virus Genomes from Orangutan Museum Specimens and 1965 Zoo Outbreak, Rotterdam, The Netherlands

Appendix

Before describing the methodological details for this study, we first want to highlight that we are working with historical DNA. Even though the DNA is only ≈60 years old, and no deamination patterns are observable (as described in more detail in the SI), we still face similar challenges to "older" ancient DNA, including short fragment length, and abundant microbial contamination.

Laboratory workflow and sequencing

The teeth obtained from orangutan skulls were ground using a MIXER MILL MM 400 before DNA was extracted (*1*), and ssDNA libraries were prepared using an ancient DNA protocol (*2*). Grinding, DNA extraction, and library preparation were carried out under clean room conditions in the specialized ancient DNA laboratory at the University of Vienna. The DNA fragments in the genomic libraries were between 51 and 117 bp long (Supplementary Figure 1), indicating that our samples were already subject to characteristic aDNA damages despite their comparatively young age.

The shotgun sequencing was performed at the Vienna BioCenter Core Facility on an Illumina NovaSeq SP SR100 XP with 100 cycles (SE).

Host genome analysis

We analyzed the shotgun sequencing data obtained without capture to confirm the authenticity of *Pongo* sp. museum specimens. We used the Mapache ancient DNA pipeline (*3*) to map the data to the orangutan reference genome due to their close genetic relationship, making the human reference genome commonly used in genomic studies of great apes, avoiding reference bias (*4*,*5*). No typical aDNA damage patterns were observed using mapDamage2.0 (*6*) for the orangutan reads for all samples, potentially due to the relatively young age of the samples. However, short fragment lengths (Supplementary Table), another typical feature of aDNA, were observed. Furthermore, we used HuConTest (*5*) to estimate contamination and found low rates of human contamination between 0.7%–1.1% at diagnostic positions in the genome, while most of reads represent authentic orangutan DNA (Supplementary Table). In a Principal Component Analysis with previously published orangutan genomes (Supplementary Figure 2; *7)*, the two individuals with sufficient endogenous DNA (7.3% for MAM1965–547 and 13.2% for MAM1965–545) clearly cluster together with Sumatran orangutans (*Pongo abelii*).

Viral phylogenomic analysis

The libraries were also captured with a myBaits custom capture kit, which targeted 99 different viral species that have been or could be associated with great apes, either as natural hosts or as spillover cases. The enriched libraries were pooled, and sequencing was performed at the Helmholtz Institute for One Health in Greifswald, Germany, on an Illumina MiniSeq.

First, the fastq files were trimmed and quality filtered with trimmomatic (*8*). Then, clumpify was used to remove duplicates (*9*). To determine the metagenomic composition of the sequenced libraries, taxonomic classification via Kraken2 (*10)*) was performed. This step checked for the presence of viral reads. Next, the quality-filtered data was mapped against a ~~the~~ monkeypox reference genome (accession number: KJ642614) with BWA (*11*), using bwa aln -n 0.04 -l 1000. A reference-based approach was chosen over a de novo approach due to the small fragment length, sequencing depth and enriched nature of the libraries, as is the norm for ancient DNA datasets. Coverage and mapping statistics were visualized using aDNA-BAMplotter (https://zenodo.org/records/5702679), several statistics were calculated, including edit distance, mapping quality, mapping quality ratio, and the percentage for 1-, 5-, and 10-fold coverage.

SNP call Rotterdam genome

The Rotterdam *Monkeypox virus* genome (Accession number: KJ642614) is most similar to the genome identified here (Figure 1C and Supplementary Figure 3), as this genome was sequenced from an animal that died during the MPXV outbreak in the Rotterdam Zoo in 1965 which was the best match to our sequences and very close in age to our animals. As our samples were, according to Museum information, from 1964, and orangutans were sick and succumbed to the viral infection, we wanted to analyze the level of sequence diversity and genome plasticity present during the outbreak. To investigate how similar the genomes are, a SNP calling was performed on using our mapping to the Rotterdam Zoo genome with freebayes (E. Garrison and G. Marth, unpub. data, https://arxiv.org/pdf/1207.3907.pdf). Filters were used to avoid low-quality calls (–report-monomorphic–min-alternate-count 5–min-coverage 5 -m 30 -F 0.9–ploidy 1), while the terminal repeats from positions 183,429 to 190,083 were masked in the genome (of note only one flanking repeat was assembled for this genome). We visually inspected the SNPs, which passed freebayes filters. Finally, two SNPs from MAM1965–0547 and MAM1965–0545 passed: a T >C transversion at position 22,950 and a deletion at position 181,693. We could not verify the presence of the T>C SNP in the other two genomes, as the position was not covered. MAM1965–0546 showed 1x coverage before and after the deletion, indicating the presence of the deletion in this genome as well.

Phylogeny

For this analysis we generated two phylogenies. First, a full genome multiple-sequence alignment-based phylogeny was built including our mappings to the Rotterdam genome, which allowed us to make use of large fraction of the genome (masking was limited to terminal repeats and informative sites were restricted to positions called in all genomes). Second, we generated a phylogeny including mapping of our genomes to the MPXV RefSeq reference from Clade I, which we limited to informative site in core genome intervals, which was required due to the divergent nature of the reference to our strains. This allowed us to control the introduction of a reference bias in our results via mapping to a closely related strain, as done in the first phylogeny.

For the first phylogeny, we used freebayes (E. Garrison and G. Marth, unpub. data) to perform a SNP call, masked the terminal repeats based on the reference genome and used the following flags to avoid low-quality calls (–report-monomorphic–min-alternate-count 5–min-coverage 5 -m 30 -F 0.9–ploidy 1) for our samples. SNPs were called for mappings to the *Monkeypox virus* Rotterdam genome (KJ642614). Overall, 1 variant remained for MAM1965–547, 1 for MAM1965–545, no variants for MAM1965–544, and no variants for MAM1965–546.

Bcftools consensus (*12*) (-a "N"–exclude 'FILTER = "LOWQUAL"') was used to obtain a consensus sequence of the two samples with the highest mean depth of coverage (19.11-fold and 9.57-fold), while calling uncovered and low-quality base calls as N. The phylogeny did not include the other two samples (MAM1965–544 and MAM1965–546) due to their low coverage.

Main phylogeny (Rotterdam Zoo reference)

For our maximum likelihood phylogeny, we included strains from Clade II and used the monkeypox virus reference genome from Clade I as an outgroup (NC_003010.1). The choice of viral genomes was based on previous publications: From Patrono et al. (*13*), we incorporated all strains from clade II and, additionally, four mammalian genomes that had over 180,000 bp sequenced. We chose ten strains representative for the viral diversity from lineage A of Clade IIb from Ndodo et al. (*14)* and one genome from the 2022 outbreak in lineage B1 of Clade II. A multiple-sequence alignment with a total of 43 genomes, including our two higher coverage genomes, was performed via MAFFT (*15*). A recombination check was performed via RDP4 (*16*) using the PHI test, which showed no evidence of recombination within the alignment. For the phylogeny, the alignment was filtered for positions with 100% coverage, which left 138,240 sites, including 2,001 informative sites. A maximum likelihood phylogenetic tree was calculated using IQTree2 v1.6.12 (*17*) with 1000 nonparametric bootstrap replicates. The K3Pu+F+I model was chosen by IQTree2, and the tree was formatted in Figtree v1.4.4 (http://tree.bio.ed.ac.uk/software/Figtree/).

Phylogeny Zaire clade I reference

For this phylogeny (see Supplementary Figure 3), we used our mapping to the reference genome of MPXV (NC_003310) to generate consensi sequences for the two genomes with the

highest coverage (17.89x and 8.11x) as described above. Due to the divergent nature of the reference sequence compared to our data, we performed a core-genome alignment via parsnp (*18*) to avoid biases. For this phylogeny, the informative sites from our core-genome alignment were filtered for positions with 100% coverage, which left 939 sites for our phylogenetic analysis. A maximum likelihood phylogenetic tree was calculated using IQTree2 v1.6.12 (*17*) with 1000 nonparametric bootstrap replicates. The TVMe+ASC model was chosen by IQTree2, and the tree was formatted in Figtree v1.4.4 (http://tree.bio.ed.ac.uk/software/Figtree/).

References

1. Dabney J, Knapp M, Glocke I, Gansauge M-T, Weihmann A, Nickel B, et al. Complete mitochondrial genome sequence of a middle pleistocene cave bear reconstructed from ultrashort DNA fragments. Proc Natl Acad Sci U S A. 2013;110:15758–63. PubMed https://doi.org/10.1073/pnas.1314445110

2. Kapp JD, Green RE, Shapiro B. A fast and efficient single-stranded genomic library preparation method optimized for ancient DNA. J Hered. 2021;112:241–9. PubMed https://doi.org/10.1093/jhered/esab012

3. Neuenschwander S, Dávalos DIC, Anchieri L, da Mota BS, Bozzi D, Rubinacci S, et al. Mapache: a flexible pipeline to map ancient DNA. Bioinformatics. 2023;39:btad028. **PMID: 36637197**

4. de Manuel M, Kuhlwilm M, Frandsen P, Sousa VC, Desai T, Prado-Martinez J, et al. Chimpanzee genomic diversity reveals ancient admixture with bonobos. Science. 2016;354:477–81. PubMed https://doi.org/10.1126/science.aag2602

5. Kuhlwilm M, Fontsere C, Han S, Alvarez-Estape M, Marques-Bonet T. HuConTest: Testing human contamination in great ape samples.Genome Biol Evol. 2021 Jun 8;13(6):evab117 **PMID: 34038549**

6. Jónsson H, Ginolhac A, Schubert M, Johnson PL, Orlando L. mapDamage2.0: fast approximate Bayesian estimates of ancient DNA damage parameters. Bioinformatics. 2013;29:1682–4. PubMed https://doi.org/10.1093/bioinformatics/btt193

7. Nater A, Mattle-Greminger MP, Nurcahyo A, Nowak MG, de Manuel M, Desai T, et al. Morphometric, behavioral, and genomic evidence for a new orangutan species. Curr Biol. 2017;27:3487–3498.e10. PubMed https://doi.org/10.1016/j.cub.2017.09.047

8. Bolger AM, Lohse M, Usadel B. Trimmomatic: a flexible trimmer for Illumina sequence data. Bioinformatics. 2014;30:2114–20. PubMed https://doi.org/10.1093/bioinformatics/btu170

9. US Department of Energy Joint Genome Institute. Clumpify guide [cited 2022 Dec 23]. https://jgi.doe.gov/data-and-tools/software-tools/bbtools/bb-tools-user-guide/clumpify-guide

10. Wood DE, Salzberg SL. Kraken: ultrafast metagenomic sequence classification using exact alignments. Genome Biol. 2014;15:R46. PubMed https://doi.org/10.1186/gb-2014-15-3-r46

11. Li H, Durbin R. Fast and accurate short read alignment with Burrows–Wheeler transform. Bioinformatics. 2009;25:1754–60. PubMed https://doi.org/10.1093/bioinformatics/btp324

12. Danecek P, Bonfield JK, Liddle J, Marshall J, Ohan V, Pollard MO, et al. Twelve years of SAMtools and BCFtools. Gigascience. 2021;10:giab008. **PMID: 33590861**

13. Patrono LV, Pléh K, Samuni L, Ulrich M, Röthemeier C, Sachse A, et al. Monkeypox virus emergence in wild chimpanzees reveals distinct clinical outcomes and viral diversity. Nat Microbiol. 2020;5:955–65. PubMed https://doi.org/10.1038/s41564-020-0706-0

14. Ndodo N, Ashcroft J, Lewandowski K, Yinka-Ogunleye A, Chukwu C, Ahmad A, et al. Distinct *monkeypox virus* lineages co-circulating in humans before 2022. Nat Med. 2023;29:2317–24. PubMed https://doi.org/10.1038/s41591-023-02456-8

15. Katoh K, Standley DM. MAFFT multiple sequence alignment software version 7: improvements in performance and usability. Mol Biol Evol. 2013;30:772–80. PubMed https://doi.org/10.1093/molbev/mst010

16. Martin DP, Murrell B, Golden M, Khoosal A, Muhire B. RDP4: Detection and analysis of recombination patterns in virus genomes. Virus Evol. 2015;1:vev003. PubMed https://doi.org/10.1093/ve/vev003

17. Nguyen LT, Schmidt HA, von Haeseler A, Minh BQ. IQ-TREE: a fast and effective stochastic algorithm for estimating maximum-likelihood. Mol Biol Evol. 2015;32:268–74. **PMID: 25371430**

18. Treangen TJ, Ondov BD, Koren S, Phillippy AM. The Harvest suite for rapid core-genome alignment and visualization of thousands of intraspecific microbial genomes. Genome Biol. 2014;15:524. PubMed https://doi.org/10.1186/s13059-014-0524-x

Appendix Table. Relevant statistics, including the number of reads, endogenous DNA percentage, average read length, number of mapped reads to the human reference genome and human contamination for the shotgun sequencing data

Individual	Number of reads	Endogenous DNA percentage	Average length of reads in base pairs in bp	Number of mapped reads to the human genome	Human contamination in the human-mapped reads
MAM1965–547	18,628,292	13.21%	76.21	2,460,890	1.1%
MAM1965–545	26,527,619	7.33%	76.22	1,945,412	1.1%
MAM1965–544	34,733,360	0.23%	56.23	79,179	1.1%
MAM1965–546	48,152,067	0.30%	84.83	142,159	0.7%

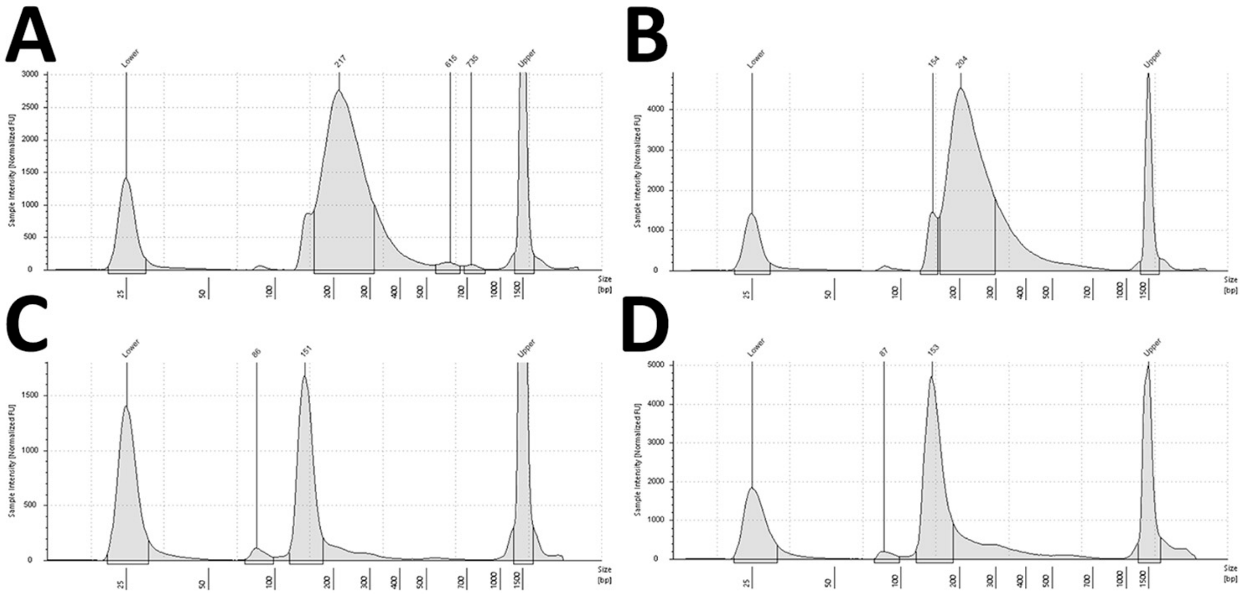

Appendix Figure 1. Size of the DNA fragments according to an automated gel electrophoresis system (TapeStation) for libraries before target-enrichment capture (A) MAM1965–0547, (B) MAM1965–0545, (C) MAM1965–0544, and (D) MAM1965–0546. The libraries include the Illumina Truseq adaptors.

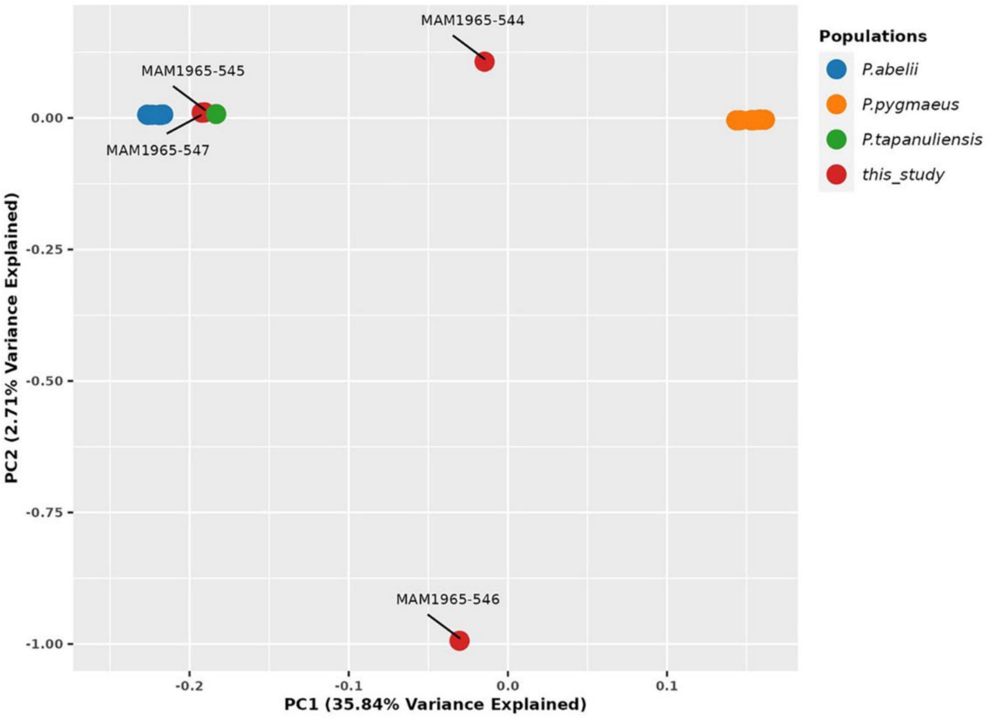

Appendix Figure 2. Principal Component Analysis (PCA) with previously published orangutan genomes. Museum samples shown in orange: two samples with sufficient endogenous DNA cluster with Sumatran orangutans (*Pongo abelii* in blue and *Pongo tapanueliensis* in red). Two samples fall in between Sumatran and Bornean orangutans (*Pongo pygmaeus* in green), most likely due to insufficient data.

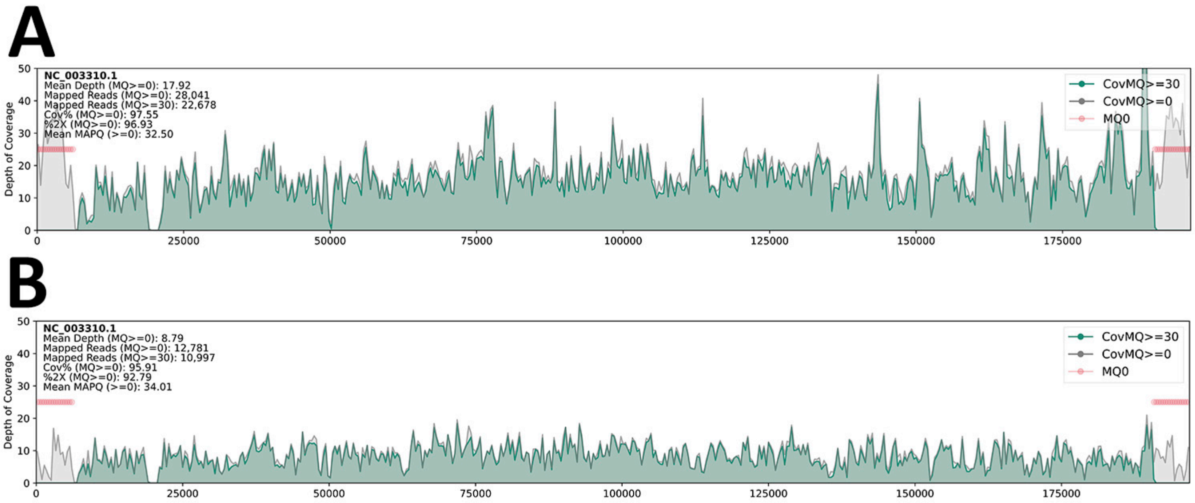

Appendix Figure 3. (A) and (B) Coverage plot for the mapping to the main Clade I *Monkeypox virus* reference sequence (ID: NC_003310). Coverage for reads with mapping quality (MQ) equal or above 30 are plotted in green, while gray areas indicate regions with reads with lower MQ. The sequence coverage is shown across the full reference genome. (A) is from sample MAM1965–0547 and (B) from MAM1965–0545.

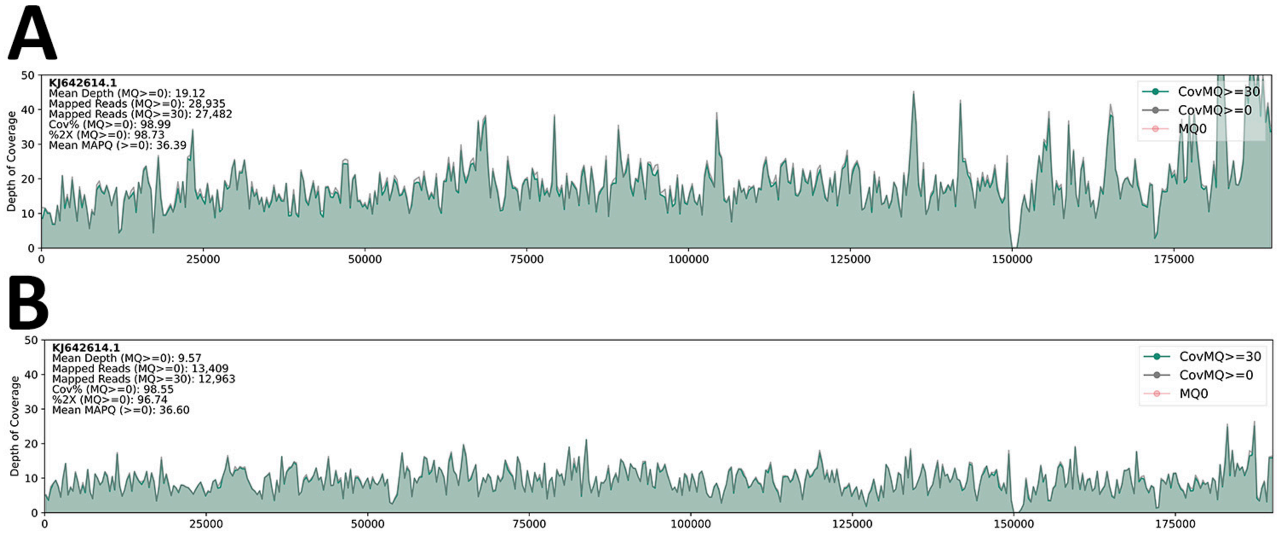

Appendix Figure 4. (A) and (B) Coverage plot for the mapping to the available Rotterdam Zoo *Monkeypox virus* genome (ID: KJ642614). Coverage for reads with mapping quality (MQ) equal or above 30 are plotted in green, while gray areas indicate regions with reads with lower MQ. The sequence coverage is shown across the full reference genome. (A) is from sample MAM1965–0547 and (B) from MAM1965–0545

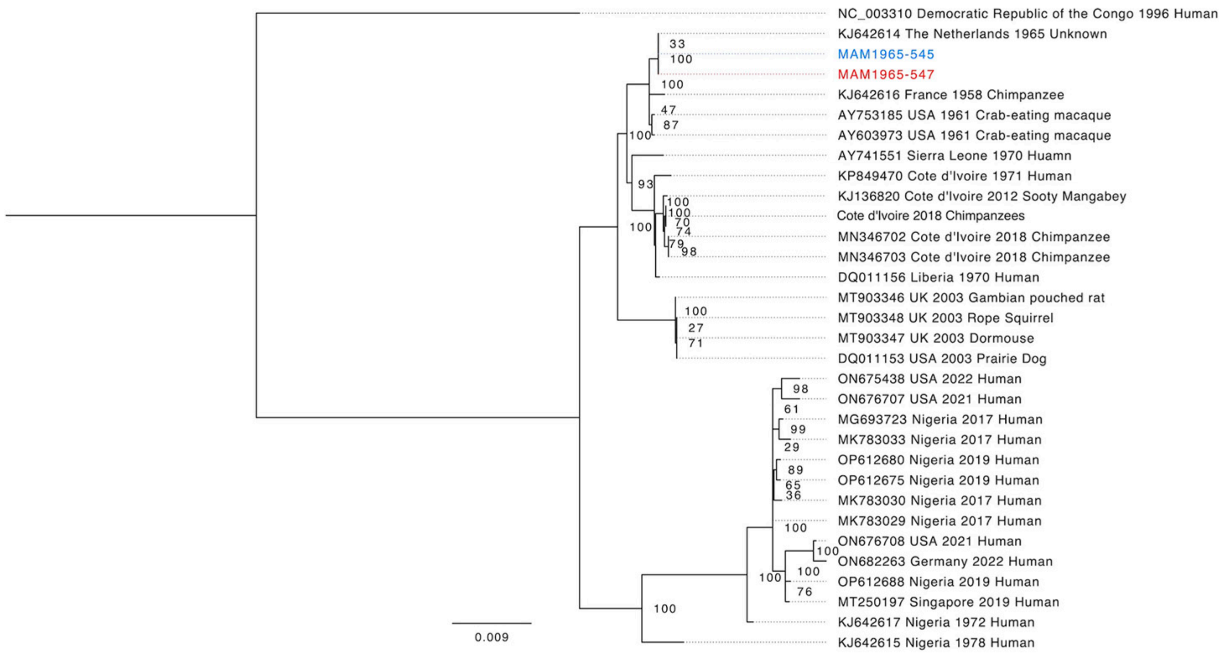

Appendix Figure 5. A core-genome phylogeny rooted on the outgroup genome NC_003310 from Clade I with the new orangutan genomes displayed in red (MAM1965–0545) and blue (MAM1965–0547). The consensus sequences for the ancient sequences are based on a mapping to the Zaire genome. The final SNPs alignment length was 939 bp. The collapsed node contains genomes from *Pan troglodytes verus* from the Cote d'Ivoire: MN346690, MN346692, MN346694 - MN346698, MN346700-MN346701.
